# Impact of early tracheostomy on resource utilization and patient outcomes in trauma ICU patients: A retrospective cohort study from southern India

**DOI:** 10.1371/journal.pone.0334938

**Published:** 2025-10-21

**Authors:** Rajesh Kamath, Sakshi Deshpande, Gwendolen Rodrigues, Tarushree Bari, Vishwajeet Singh, Sandesh Kini, Nahima Akthar, Gavi Salimath

**Affiliations:** 1 Department of Healthcare and Hospital Management, Prasanna School of Public Health, Manipal Academy of Higher Education, Manipal, Karnataka, India; 2 Department of Social and Health Innovation, Prasanna School of Public Health, Manipal Academy of Higher Education, Manipal, Karnataka, India; 3 Directorate of Online Education, Manipal Academy of Higher Education, Manipal, Karnataka, India; 4 Department of Pediatrics, Kasturba Medical College, Manipal Academy of Higher Education, Manipal, Karnataka, India; 5 Department of Paediatric Nursing, KAHER Institute of Nursing Sciences, KLE University, Belagavi, Karnataka, India; University of Florida Jacksonville, UNITED STATES OF AMERICA

## Abstract

**Introduction:**

A tracheostomy is an important intervention for trauma patients referred to intensive care units (ICUs). Trauma patients often require prolonged intubation; timing of tracheostomy remains debated.The purpose of this study is to determine the impact of early tracheostomy on critical metrics such as mechanical ventilation duration, ICU length of stay (LOS) and ventilator acquired pneumonia (VAP) in trauma patients in ICU settings.

**Methods:**

We conducted a retrospective cohort study of 383 trauma patients who underwent tracheostomy in a tertiary teaching hospital ICU (January 2018–December 2022). Inclusion: trauma patients with temporary tracheostomy; Exclusion: permanent tracheostomies. Early tracheostomy (ET) was defined as ≤7 days of mechanical ventilation, late (LT) as >7 days. The dataset includes demographic information, Acute physiology and chronic health evaluation II score, Simplified acute physiology score II, Glasgow coma scale score, Injury severity Score, type and cause of injuries, ICU outcomes, length of stay and rates of ventilator-associated pneumonia (VAP). Data were analyzed using Mann–Whitney U and Chi-square tests; significance at p < 0.05.. The study involved a comparison of the duration of mechanical ventilation, ICU LOS, VAP rates and extubation trials between patients who underwent ET and LT.

**Results:**

Of the 804 patients who underwent tracheostomies from January 2018 to December 2022, 383 were trauma patients and were included in the study. There were no significant differences between the two groups in terms of age, sex, Acute physiology and chronic health evaluation II score, Simplified acute physiology score II and Injury severity score. The incidence of VAP was lower in the ET cohort (15.9%) than in the LT cohort (47.4%). The percentage of extubation trials was found to be higher in the LT cohort (43.1%) than in the ET cohort (9.3%), resulting in prolonged ICU LOS. Patients with an ET had a significantly shorter ICU LOS median of 15 days (IQR 13,17) and a mechanical ventilation median of 13 days (IQR 11,14) than LT patients who had an ICU LOS median of 33 days (IQR 30,36) and a mechanical ventilation median of 31 days (IQR 27,33) respectively.

**Conclusion:**

Implementing an early tracheostomy protocol for trauma patients in the ICU is associated with a decreased incidence of VAP, shorter duration of mechanical ventilation and shorter ICU LOS while maintaining consistent ICU and hospital outcomes. The adoption of a standardized approach to perform early tracheostomy helps in improving resource utilization and patient outcomes in trauma patients.

## Introduction

Trauma is a major public health concern and one of the leading causes of intensive care unit (ICU) admissions worldwide. The World Health Organization (WHO) estimates that road traffic accidents (RTAs) result in more than 1.25 million deaths annually [1]. In India, trauma accounts for more than one-third of unnatural fatalities, with a significant economic impact estimated at 2–3% of the country’s gross domestic product (USD 7.2 to 10.8 billion) each year [2]. Despite having only 1% of the world’s motorized vehicles, India contributes to nearly 11% of global RTAs [3]. Annually, India records around 450,000 RTAs, leading to the loss of 150,000 lives [4], and RTAs alone account for 60% of traumatic brain injury (TBI) cases in the country [5]. This growing burden, compounded by rapid highway expansion and increasing passenger vehicle use [5–8], underscores the pressing need for efficient trauma care strategies. Globally, traumatic injuries are responsible for 5.8 million deaths each year, accounting for 10% of overall mortality [9].

A substantial proportion of critically injured trauma patients require airway protection and mechanical ventilation. It is estimated that 30–40% of ICU trauma patients undergo endotracheal intubation, and among them, approximately 10–15% require a tracheostomy for prolonged ventilatory support [2,5–7]. While tracheostomy offers several advantages such as reducing oropharyngeal irritation, maintaining airway patency, decreasing ventilator-associated complications, and facilitating weaning from sedatives and ventilators [10] it is not without risks. The procedure itself can cause complications, and there remains ongoing debate about the optimal timing of its implementation [11]. Early tracheostomy (ET) may benefit certain subgroups, such as patients with severe TBI or significant head and neck trauma, by reducing ventilator duration and ICU stay [[10,12–17,18]]. In contrast, patients with prolonged respiratory failure without head or neck injuries are harder to predict for tracheostomy needs, making timing decisions more complex.

The clinical impact of ET has been explored extensively. Studies have reported that ET is associated with reduced ventilator-associated pneumonia, lower ICU average length of stay (ALOS), decreased sedation requirements, and improved patient comfort and communication [4,10,14–16]. Rizk et al. demonstrated that patients in late tracheostomy (LT) cohorts experienced longer ICU stays compared to those in ET cohorts [19]. Similarly, patients undergoing ET within 7 days of intubation had shorter ICU stays, lower mortality, and reduced duration of mechanical ventilation than those who underwent LT [19,20]. While some systematic reviews and meta-analyses found no survival benefit [21], others including Cochrane and large pooled analyses have shown reduced mortality and ICU ALOS with ET [15,16,17,22]. Recent studies have also highlighted its potential role during the COVID-19 pandemic, where ET facilitated faster ventilator weaning and improved ICU resource utilization [23]. Despite this evidence, most data come from high-income settings, and limited literature exists on trauma patients in Indian tertiary care hospitals. This study addresses this gap by evaluating the impact of ET versus LT on patient outcomes and resource utilization among trauma patients admitted to a teaching hospital ICU in Karnataka, India.

## Methodology

### Study design and setting

This was a retrospective observational study conducted at a tertiary care teaching hospital in coastal Karnataka, India. The hospital is a 2,000-bedded, NABH-accredited facility that provides healthcare services to both domestic and international patients. Data were collected from the Intensive Care Unit (ICU) and the Medical Records Department. The study examined trauma patients admitted between January 2018 and December 2023, and data extraction was performed from October 30 to December 30, 2023.

### Study population

We included adult trauma patients (≥18 years) who underwent a temporary tracheostomy during their ICU stay. Patients with permanent tracheostomies (e.g., for chronic head and neck conditions) were excluded. Patients who required re-intubation after extubation were included in the analysis if they subsequently underwent a tracheostomy within the same admission.

### Tracheostomy timing

Patients were classified into two groups based on timing of the procedure:

- Early tracheostomy (ET): performed within 7 days of endotracheal intubation.
- Late tracheostomy (LT): performed after 7 days of intubation.

### Tracheostomy technique

Where available, details of the procedure type (surgical vs. percutaneous) and location (bedside in ICU vs. operating room) were recorded from operative notes. Since this information was not consistently documented for all patients, it is reported as a limitation of the study.

### Data collection

A validated proforma was used to collect information from medical records. Variables included demographic data (age, gender), mechanism and type of injury, clinical scores (GCS, SAPS II, APACHE II, ISS), extubation trials, incidence of ventilator-associated pneumonia (VAP), duration of mechanical ventilation, tracheostomy-related complications (e.g., bleeding, infection, accidental decannulation), ICU and hospital length of stay (LOS), and in-hospital mortality. Patients were also stratified by indication for intubation (e.g., traumatic brain injury, chest trauma, polytrauma). Records with >20% missing key variables (timing of tracheostomy, LOS, outcome measures) were excluded. Data on readmission rates and time to decannulation were intended to be collected; however, these parameters were not consistently documented in patient records during the study period. As such, they could not be reliably included in the analysis.

### Sample size

A total of 804 medical records of trauma patients who underwent tracheostomy between January 2018 and December 2023 were reviewed.

### Statistical analysis

Data were analyzed using Jamovi version 2.3.18. Continuous variables were summarized using means, medians, and interquartile ranges, while categorical variables were presented as frequencies and percentages. Since the data were not normally distributed, comparisons between ET and LT groups were performed using the Mann–Whitney U-test for continuous variables and the Chi-square test for categorical variables. A p-value <0.05 was considered statistically significant.

### Ethical approval

Permission to access files to conduct the retrospective record based study was given by the Medical Superintendent of Kasturba Hospital, Manipal subject to IEC approval. The study was approved by the Kasturba Medical College and Kasturba Hospital Institutional Ethics Committee (IEC) of Manipal Academy of Higher Education, Manipal, Karnataka, India (Approval No. IEC2: 545/2023, dated November 27, 2023). Informed consent is not applicable as the study involves secondary analysis of anonymized medical records. Data for this study was obtained from a 5 year retrospective ICU database that included information on patients who underwent tracheostomy with unique identification numbers given to each patient to maintain anonymity. All datasets were de-identified and assigned unique identification numbers to ensure confidentiality. No minors were included in this study.

## Results

**Fig 1:**
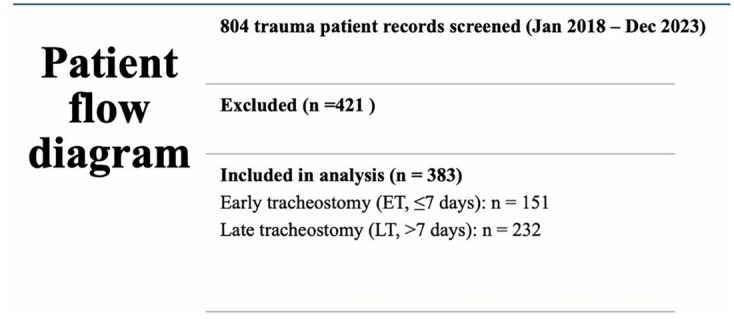
Flow diagram of patient selection.

A total of 804 patients underwent tracheostomy during the study period (January 2018 – December 2023), of which 383 trauma patients met the inclusion criteria as shown in Fig 1.

The demographic and baseline clinical features of the study population are summarized in Table 1. The early tracheostomy (ET) and late tracheostomy (LT) groups were comparable in terms of age, sex distribution, Injury Severity Score (ISS), Simplified Acute Physiology Score II (SAPS II), and Acute Physiology and Chronic Health Evaluation II (APACHE II) scores, with no statistically significant differences observed.

**Table 1:** Demographic and baseline characteristics of trauma patients by tracheostomy timing (ET vs LT).

Variables	Mean	Median	SD	Min	Max	Shapiro-Wilk (p- value)
Age	36.78	36	8.54	11	51	<.001
APACHE Il score	25.28	25	3.02	18	35	<.001
SPAS Il score	31.77	32	6.67	20	45	<.001
GCS score	7.76	8	1.21	5	10	<.001
ISS score	15.93	16	3.44	9	25	<.001
Venti days before T	9.22	11	4.25	2	16	<.001
Days (ICU – T)	9.72	12	4.64	2	17	<.001
Days (T to W)	14.23	15	5.50	5	32	<.001
Days (T – ICU discharge)	16.17	17	5.59	5	38	<.001
Total MV days	23.46	26	9.28	7	44	<.001
ICU LOS	25.90	29	9.62	8	51	<.001

Compared with the LT group, patients in the ET group had a significantly lower incidence of ventilator-associated pneumonia (VAP) (15.9% vs. 47.4%). They also experienced shorter median ICU length of stay (15 days [IQR 13–17] vs. 33 days [IQR 30–36]) and reduced duration of mechanical ventilation (13 days [IQR 11–14] vs. 31 days [IQR 27–33]) as shown in Table 2.

**Table 2:** Clinical outcomes of trauma patients undergoing early vs. late tracheostomy.

Variables	Early Tracheostomy (<=7 days)	Late Tracheostomy (>= 7days)	p- value
**Age (in years)**	37.31 ± 8.09	36.44 ± 8.83	0.545
**APACHE II score**	25.40 ± 3.12	25.20 ± 2.95	0.810
**SAPS II score**	32.06 ± 6.18	31.57 ± 6.97	0.399
**ISS score**	15.72 ± 3.66	16.07 ± 3.29	0.364
**GCS score**	7.60 ± 1.27	7.86 ± 1.16	0.056
**Ventilation days before Tracheostomy**	4.25 ± 1.24	12.47 ± 1.47	<.001
**Days from ICU admission to tracheostomy**	4.25 ± 1.24	13.28 ± 1.52	<.001
**Days from tracheostomy to weaning.**	8.56 ± 1.68	17.92 ± 3.68	<.001
**Days from tracheostomy to ICU discharge**	10.70 ± 2.36	19.73 ± 3.97	<.001
**Total duration of mechanical ventilation days**	12.81 ± 2.05	30.39 ± 4.15	<.001
**ICU LOS**	14.95 ± 2.59	33.03 ± 4.38	<.001

Extubation trials were more frequent in the LT group (43.1%) compared to the ET group (9.3%). Additional subgroup analyses stratified by indication for intubation (e.g., traumatic brain injury, chest trauma, polytrauma) are presented in Table 3. Tracheostomy-related complications (such as bleeding, infection, accidental decannulation) and mortality outcomes are also included.

**Table 3:** Subgroup analysis of outcomes stratified by indication for intubation.

Variables	Early tracheostomy (<=7 days)	Late tracheostomy (>= 7days)	P value
**Age**			
**Male**	32.4% (124)	44.4% (170)	<0.001
**Female**	7% (27)	16.2% (62)	
**VAP**			
**Yes**	6.3% (24)	28.7% (110)	<0.001
**No**	33.2% (127)	31.9% (122)	
**Extubation trials** percentage (patient counts)	9.3% (14)	26.1% (100)	<0.001
***Cause of injury*** percentage (patient counts)			
RTA	27.2% (104)	41.8% (160)	>0.05
Falls	8.1% (31)	14.9 (57)	
Sports	2.6% (10)	2.1% (8)	
Industrial	1.6% (6)	1.8% (7)	
***Types of injuries*** percentage (patient counts)			
Head	19.3% (74)	41.5% (159)	<0.001
Maxillofacial	13.6% (52)	6.3% (24)	
Spinal	2.3% (9)	5.5% (21)	
Abdominal	2.6% (10)	4.7% (18)	
Chest	1.6% (6)	2.6% (10)	

**Fig 2:**
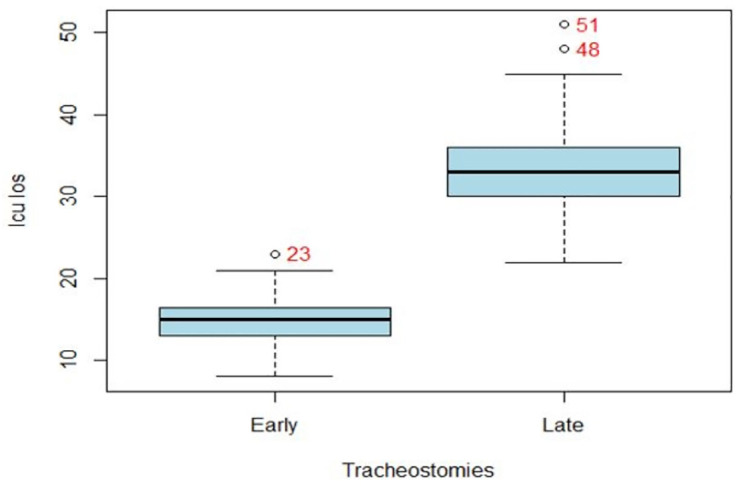
Distribution of patients Tracheostomy correspondnig ICU LOS.

**Fig 3:**
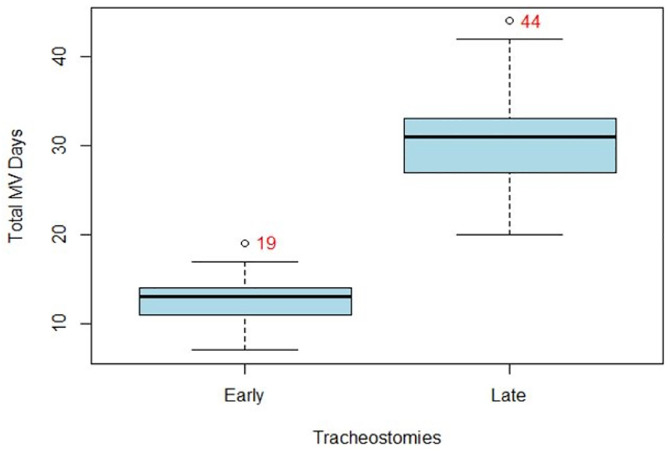
Distribution of patients by Tracheostomy and mechanical ventilation days.

Figs 2 and 3 illustrate that patients with an ET had a significantly shorter ICU LOS median of 15 days (IQR 13,17) and mechanical ventilation days’ median of 13 days (IQR 11,14) than the patients with a LT median of 33 days (IQR 30,36) and median of 31 days (IQR 27,33) and the p value indicates the same (p < 0.001) for both the cases.

Overall, ET was associated with fewer pulmonary complications and markedly better resource utilization, while maintaining comparable survival outcomes between groups. Readmission rates and time to decannulation could not be reported, as these data were not consistently captured in the dataset and did not meet the study’s quality threshold for inclusion.

## Discussion

A tracheostomy is a frequently performed intervention in the ICU for patients requiring prolonged mechanical ventilation. A tracheostomy is a frequently performed intervention in the ICU for patients requiring prolonged mechanical ventilation. Traditionally, the procedure has often been delayed until **after 10 days of intubation**, although considerable variation exists across institutions and studies [24,25,26]. The role of Early Tracheostomy (ET) in improving patient outcomes remains debated, but growing evidence supports its utility in critically ill trauma patients.

### Timing, ICU stay, and mechanical ventilation

Our findings align with previous studies demonstrating that ET is associated with reduced ICU length of stay (LOS) and fewer days on mechanical ventilation [27,28]. In our cohort, ET patients had a median ICU LOS of 15 days compared to 33 days in the Late Tracheostomy (LT) group, mirroring findings by Kang et al., who reported median stays of 21 versus 35 days 24. Romero et al. and Alali et al. also observed shorter ventilation duration and ICU stays in patients who underwent ET following traumatic brain or spinal cord injury [27,29]. These benefits are likely attributable to earlier sedation weaning, improved secretion management, and earlier initiation of rehabilitation.

### Ventilator-Associated Pneumonia (VAP)

Another significant observation in our study was the lower incidence of VAP in the ET cohort. This supports evidence from randomized controlled trials and systematic reviews that late tracheostomy (>7 days) is linked with higher VAP risk compared to earlier procedures [23,28]. By reducing the duration of endotracheal intubation, ET may decrease airway colonization and improve pulmonary hygiene, which helps explain the lower VAP rates observed.

### Mortality outcomes

Consistent with earlier reports, our study did not demonstrate significant mortality differences between ET and LT [27,29]. Previous analyses have shown mixed results: while Romero et al. found no impact on ICU mortality [27], Hsu et al. observed higher mortality and weaning failure in patients undergoing tracheostomy after 21 days [30]. Conversely, Keenan et al. noted increased mortality when tracheostomy was performed within 10 days in trauma patients without head injuries [13,31]. These conflicting findings highlight the complex interplay of injury severity, comorbidities, and patient selection in determining outcomes.

### Resource utilization

Our results underscore the potential of ET to optimize ICU resources. Shorter ICU stays, reduced ventilator use, and fewer VAP cases translate into improved bed turnover, reduced antimicrobial use, and lower staffing demands. Although we did not perform a cost analysis, prior studies have shown substantial cost savings when tracheostomy is performed earlier [32]. In resource-constrained settings like India, such benefits are particularly valuable, as they can enhance ICU throughput without compromising care quality[33].

### Patient characteristics and selection bias

We observed that patients with low Glasgow Coma Scale (GCS) scores or severe head and maxillofacial injuries were more likely to undergo ET. This reflects common clinical practice, where clinicians prioritize early airway stabilization in patients with poor neurological prognosis or airway compromise. Goettler et al. similarly emphasized the importance of early intervention in trauma patients with low GCS to improve both outcomes and efficiency [34].

Although direct cost data were not available in this study, ICU length of stay and ventilator days are well-recognized drivers of hospital expenditure. In our cohort, the ET group had an 18-day shorter median ICU stay compared to LT patients. Prior studies have estimated daily ICU costs for trauma patients to range from USD 1,200–2,500, depending on case complexity and resource intensity [32]. Extrapolating from these estimates, the observed reduction in LOS in the ET cohort suggests substantial potential cost savings. While we did not perform micro-costing, future prospective studies incorporating detailed financial data could help quantify these benefits more precisely and strengthen the economic case for adopting early tracheostomy strategies.

### Limitations

This study has several limitations. First, its retrospective, single-center design limits generalizability. Second, data on the exact reasons for early versus late tracheostomy decisions (e.g., family consent delays, clinical uncertainty) were not available and thus could not be analyzed. Third, although we recorded complications when documented, inconsistent reporting may have led to underestimation. Finally, cost data were not included, which would have strengthened the analysis of resource utilization. Another limitation of this study is the absence of reliable data on readmission rates and time to decannulation. These outcomes were not consistently documented in medical records and were therefore excluded from analysis. Future prospective studies should include these measures to provide a more comprehensive assessment of long-term outcomes following tracheostomy.

### Strengths and novelty

Despite these limitations, our study adds important insights to the debate on tracheostomy timing. Few Indian studies have specifically examined trauma populations in the ICU, where the burden of road traffic accidents and critical injuries is particularly high. By demonstrating that ET is associated with reduced ICU LOS, shorter ventilation duration, and fewer VAP cases, our study highlights the potential for ET to improve both patient outcomes and resource use in a resource-limited, high-burden context.

## Supporting information

S1 File Tracheostomy data. (XLSX)
